# Curricula and resources related to social entrepreneurship and public health innovation within schools of public health in the United States

**DOI:** 10.3389/fpubh.2024.1354787

**Published:** 2024-02-09

**Authors:** Ingeborg Hyde, Kaveh Khoshnood, Teresa Chahine, Fatema Basrai

**Affiliations:** ^1^InnovateHealth Yale, New Haven, CT, United States; ^2^School of Public Health, Yale University, New Haven, CT, United States; ^3^School of Management, Yale University, New Haven, CT, United States

**Keywords:** public health innovation, public health entrepreneurship, design thinking, innovation and entreprenuership, public health education

## Abstract

This paper examines the current state of social innovation and entrepreneurship programming, courses, and centers within schools of public health through a survey data analysis. This report presents a cross-sectional survey conducted among faculty members of public health schools in the United States. The survey aims to determine the availability and current state of student-centered programs and courses centered around social innovation and entrepreneurship within schools of public health. Insights were drawn from 19 professionals across 15 schools of public health. Uncertainties surround the sustainability of current programs, with insufficient funding, human resources, and the need to teach more pressing topics identified as the most significant obstacles. Key areas identified as opportunities for growth were faculty engagement, expertise, and funding to expand more structured programming.

## Introduction

In the last decades, there has been a rising interest in social innovation and social entrepreneurship across many disciplines (1). According to The Organization for Economic Co-operation and Development, social innovation refers to “the design and implementation of new solutions that imply conceptual, process, product, or organizational change, which ultimately aim to improve the welfare and wellbeing of individuals and communities” (2). Innovative thinking has been used in a variety of fields to promote efficiencies and social change, ranging from education to healthcare and beyond. Scholars have also cited that ventures rooted in social innovation tend to have lower cost structures and operate more efficiently due to their blend of market and nonmarket approaches (3).

Currently, there is not an accurate gauge as to which schools of public health offer courses or programming based on social innovation and entrepreneurship. While various published papers have outlined the strong connection between public health, social innovation, and entrepreneurship, to our team’s knowledge, there is not a published paper that explores the current status of programming and curricula of this space within schools of public health. The purpose of this study is to gain a better understanding of which schools offer classes and programming with a focus in “social entrepreneurship” and “innovation.” This study can help public health professionals and educators recognize if “social entrepreneurship” and “innovation” are rising areas of interest. Lastly, this study will be helpful in determining whether and how schools of public health can invest resources in the potentially growing study area of public health innovation (4).

This study is being conducted out of InnovateHealth Yale (IHY), a program founded in 2013 and housed within the Yale School of Public Health that supports the creation of innovative solutions to challenges in public health and education for underserved communities in the United States and low-resource countries; IHY is the first program of its kind housed within a school of public health in the United States (5).

## Methodology

### Data collection

In this paper, a cross-sectional study, in the form of a survey, was conducted with faculty members of public health schools. Participants designated the extent to which their schools of public health offer student-facing social innovation and entrepreneurship-based courses and/or programs. The survey was distributed electronically, with potential participants receiving an email invitation in the period from early February 2023 through April 2023.

### Recruitment

Participants were sought through the Council on Education for Public Health website, individual school websites, social media, and through word of mouth. To enhance the reach and diversity of respondents, participants were encouraged to share the survey with their colleagues within schools of public health. This snowball sampling approach aimed to capture a broad spectrum of perspectives within the public health community.

### Data collection

The survey is comprised of 20 questions, spanning from gathering demographic information to delving into more intricate details about innovation-based programming and curricula. These inquiries are strategically organized to explore three primary domains: (1) structural additions to public health innovation and entrepreneurship education, such as centers or programming; (2) courses related to public health innovation and entrepreneurship curriculum; (3) future plans to expand public health innovation and entrepreneurship.

## Results

**Figure 1:**
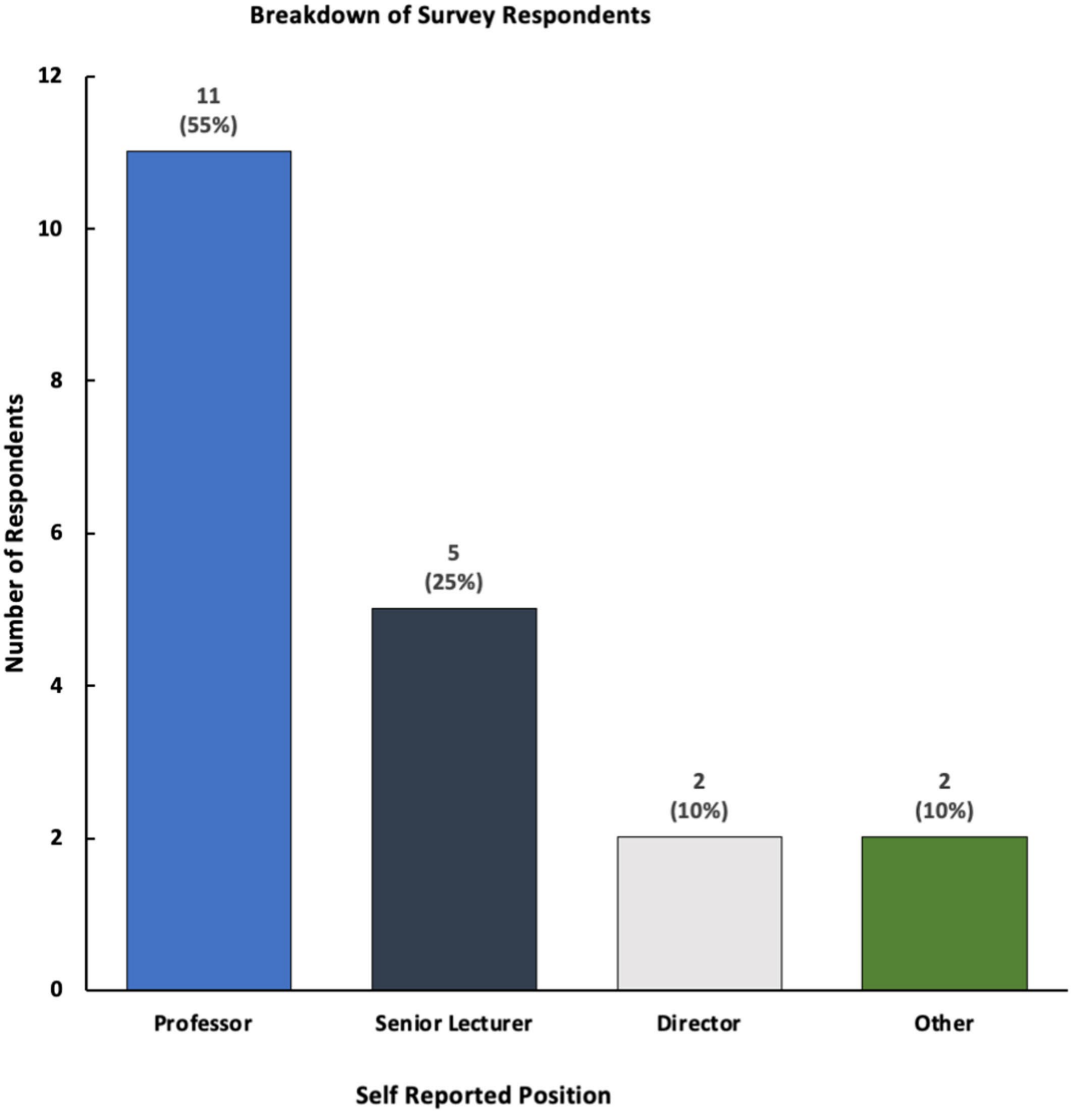
Breakdown of respondents.

**Figure 2:**
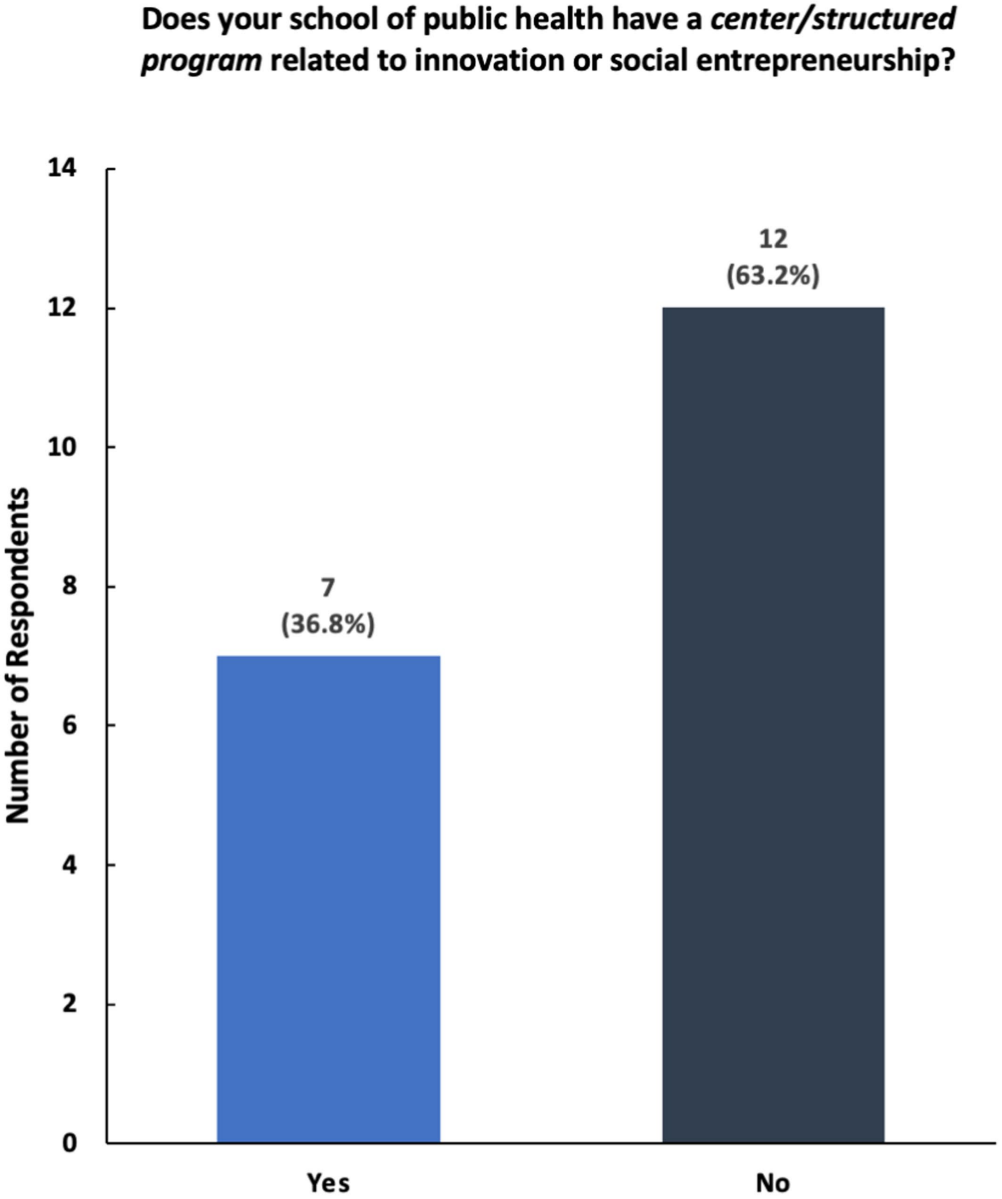
Breakdown of responses to the survey question—“Does your school of public health have a center/structured program related to innovation or social entrepreneurship?”

Our survey collected the insights of 19 professionals who work at 15 schools of public health across the United States; our team approached 71 professionals, creating a 26.8% (*n* = 19) response rate (Figure 1). Although fewer than 40% (*n* = 7) of respondents reported having a center or structured program dedicated to these areas, a majority of respondents stated that their schools of public health offer courses and programming related to innovation or social entrepreneurship (Figure 2).

**Figure 3:**
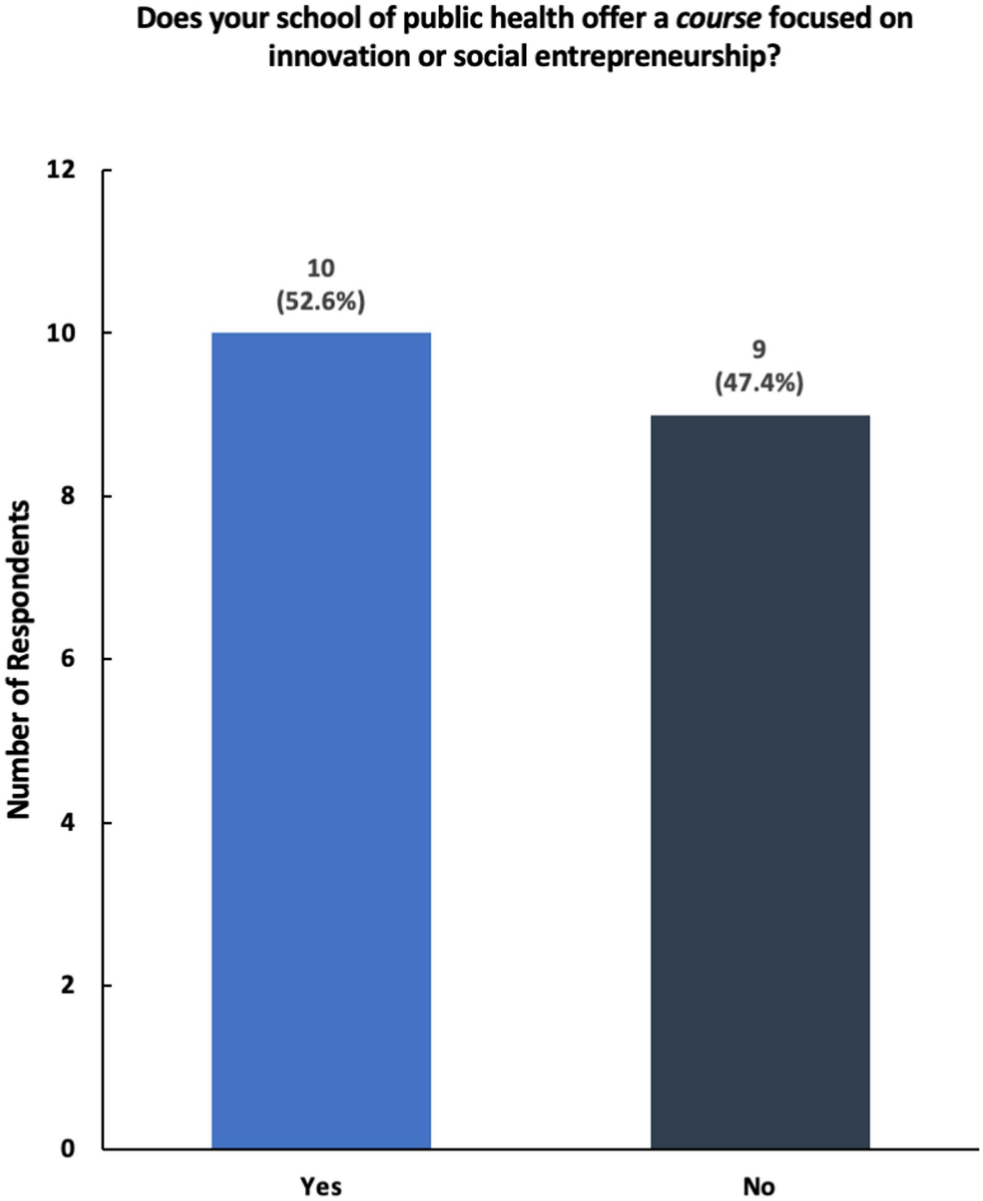
Breakdown of responses to the survey question—“Does your school of public health offer a course focused on innovation or social entrepreneurship?”

**Figure 4:**
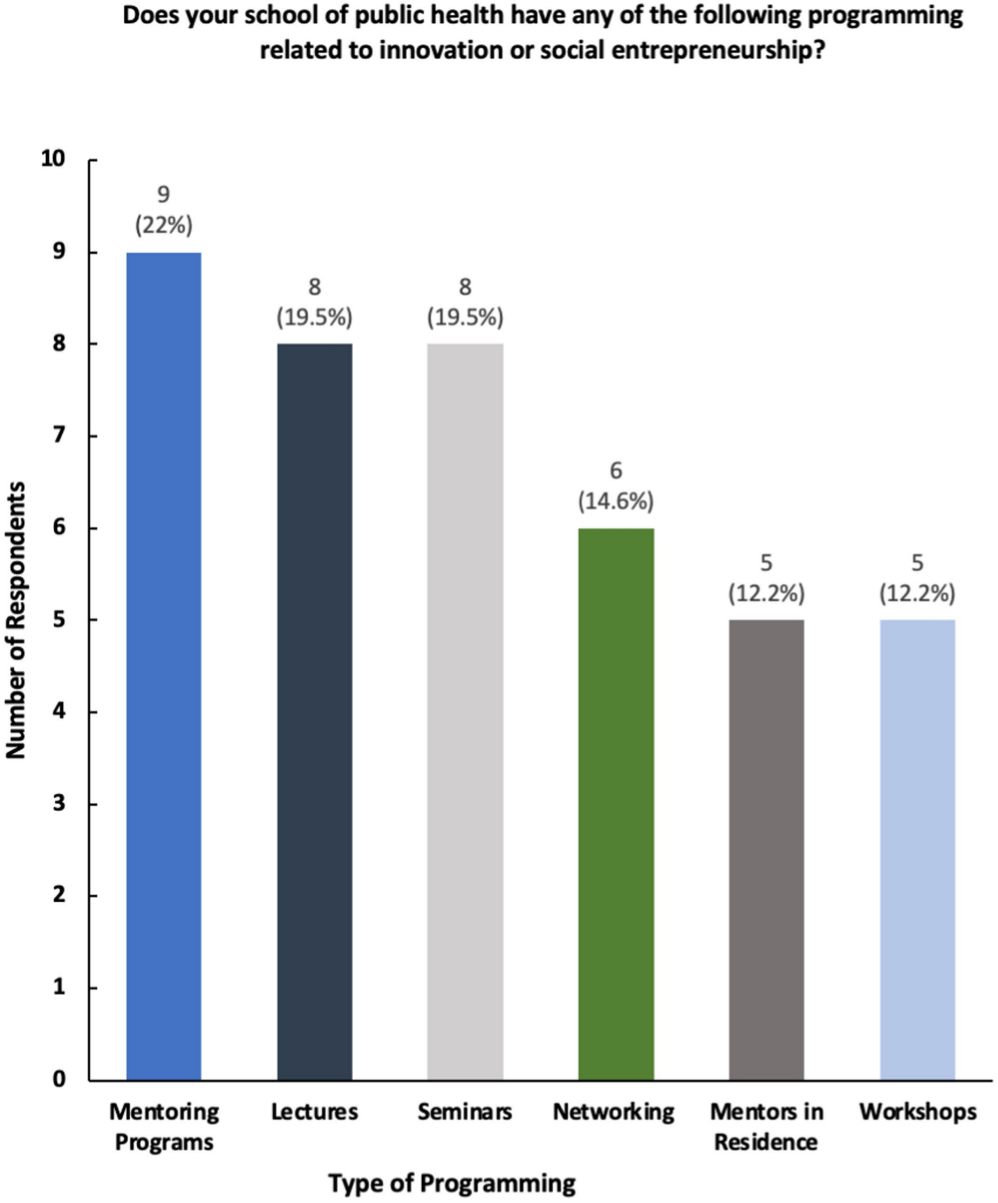
Breakdown of innovation-based programming.

**Figure 5:**
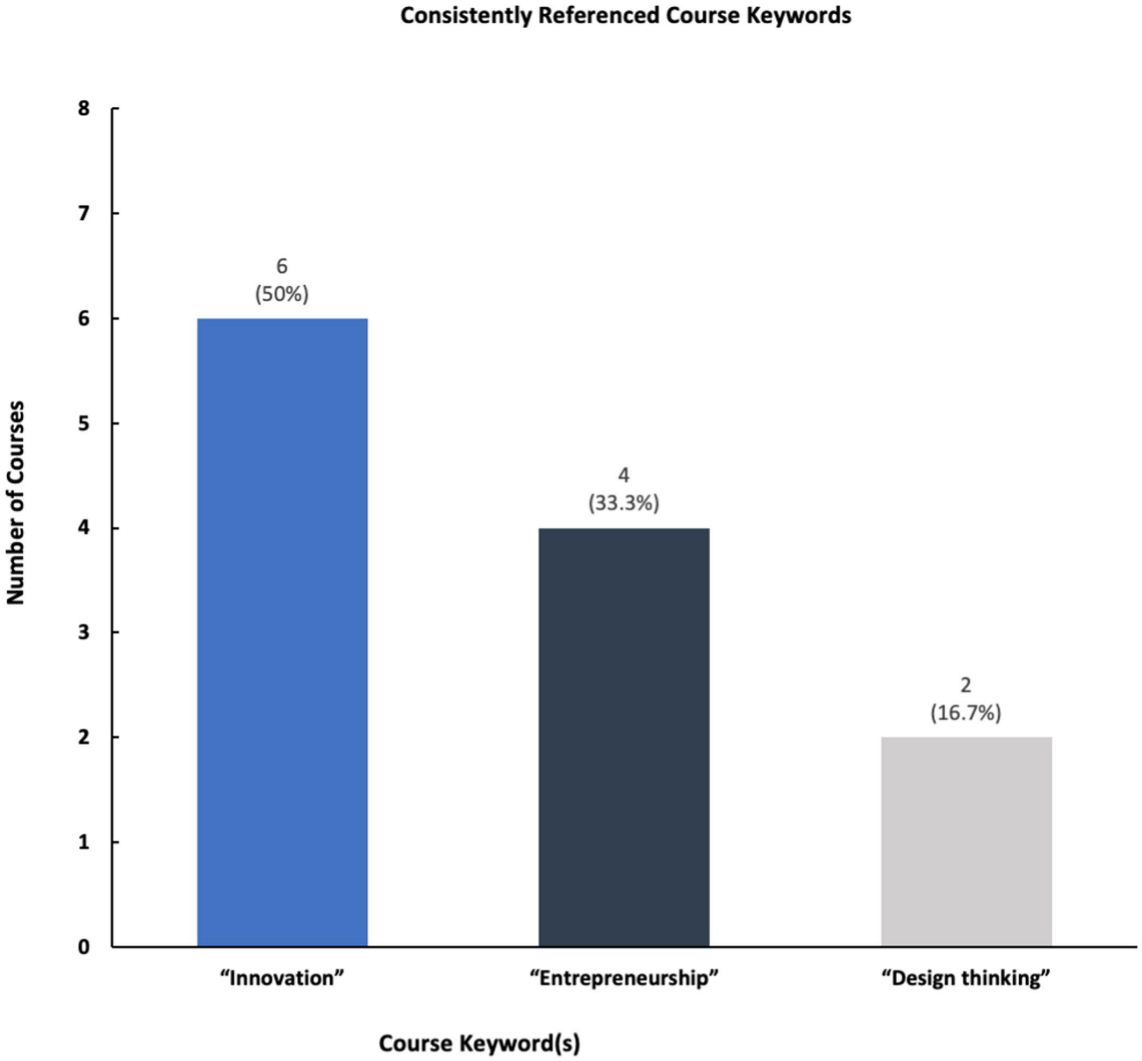
Number of consistently referenced course keywords.

**Figure 6:**
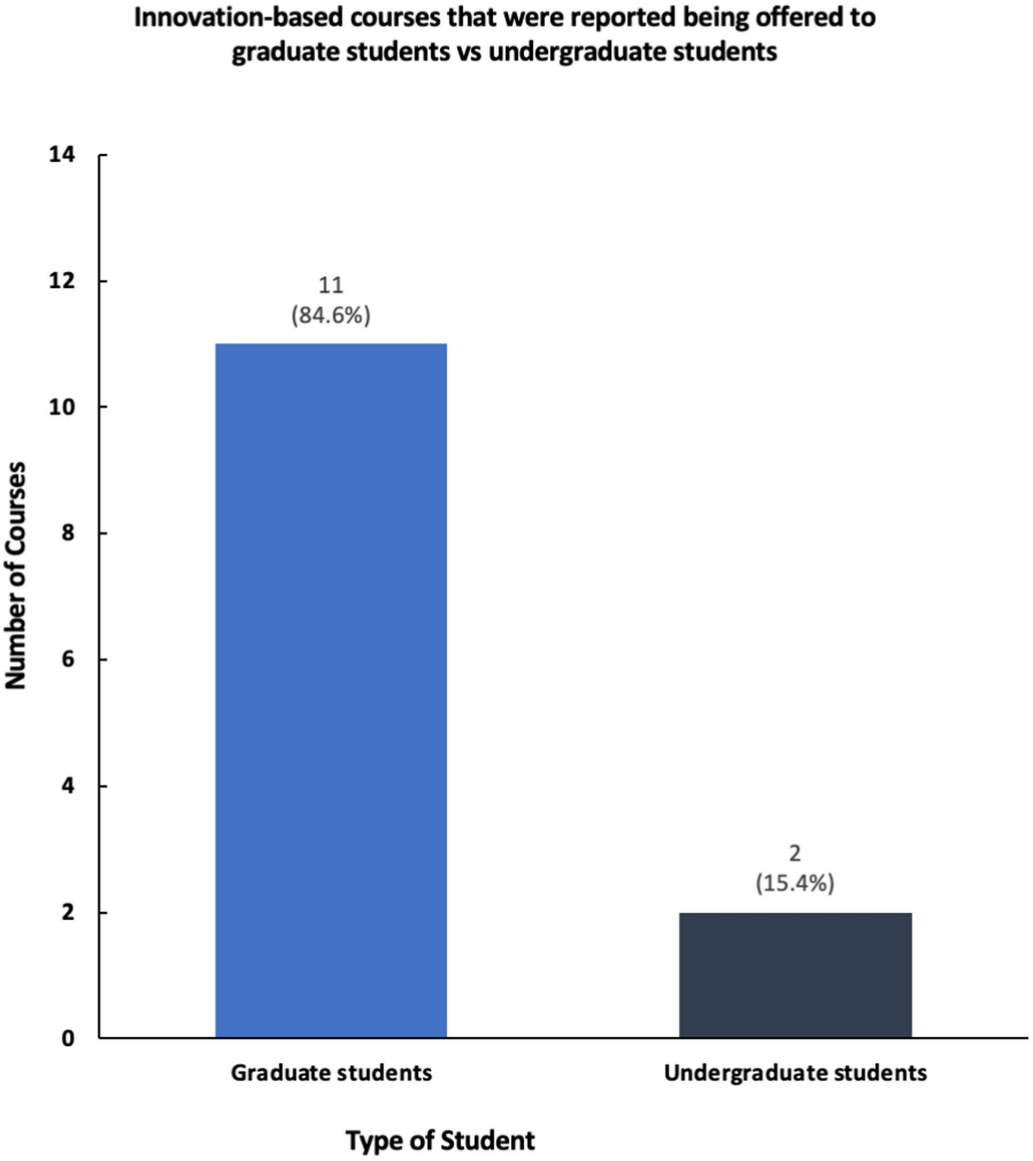
Comparison of innovation-based course offerings by student type.

The results show that while a majority of participating universities and colleges offer courses and programming in social innovation and entrepreneurship, only a select number of schools of public health have structured centers dedicated to these topics (Figures 2, 3). Furthermore, schools reported having a variety of innovation-based programming—ranging from mentoring programs, seminars, and networking opportunities (Figure 4). The reported courses in this space have the following consistently referenced keywords in their titles: “innovation,” “entrepreneurship,” and “design thinking” (Figure 5). The survey also captured the sentiment that an overwhelming number of resources are available for primarily graduate students at the observed schools of public health, including graduate-only certificates that study the intersection of public health and social innovation (Figure 6). “*There’s an appetite—especially from students. We’ve enrolled more students in our [graduate Certificate in Innovation] than we anticipated,*” responded a public health professor.

**Figure 7:**
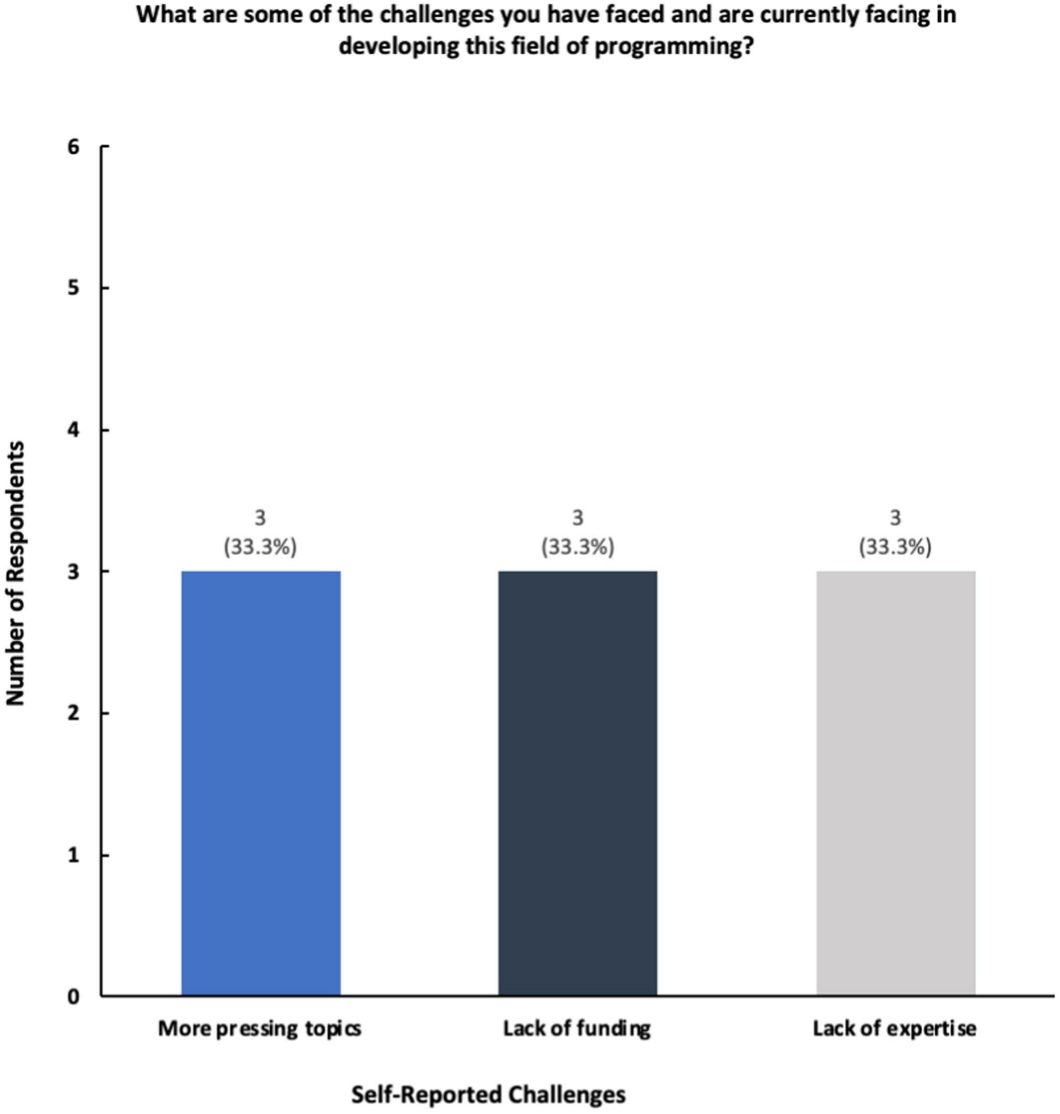
Breakdown of challenges in developing in novation-based programming.

Many respondents cited lack of faculty engagement, experts, and funding as the primary reasons as to why more structured programming cannot be further built out (Figure 7). One professor commented the following when referring to challenges in building out programming and curriculum within the public health innovation space: “*Insufficient funding or resources to support sustainable, high-quality programming for faculty and students; there is little incentive for faculty to explore this space with other competing responsibilities and priority*.” Another staff member mentioned, “*Strong interest in innovation however I do not know as there is funding available specifically for more innovation programming right now*.” However, many participants noted that their students have access to university-wide innovation and entrepreneurship centers, accelerators, and programming that aims to help students create social impact, including in the healthcare space.

**Figure 8:**
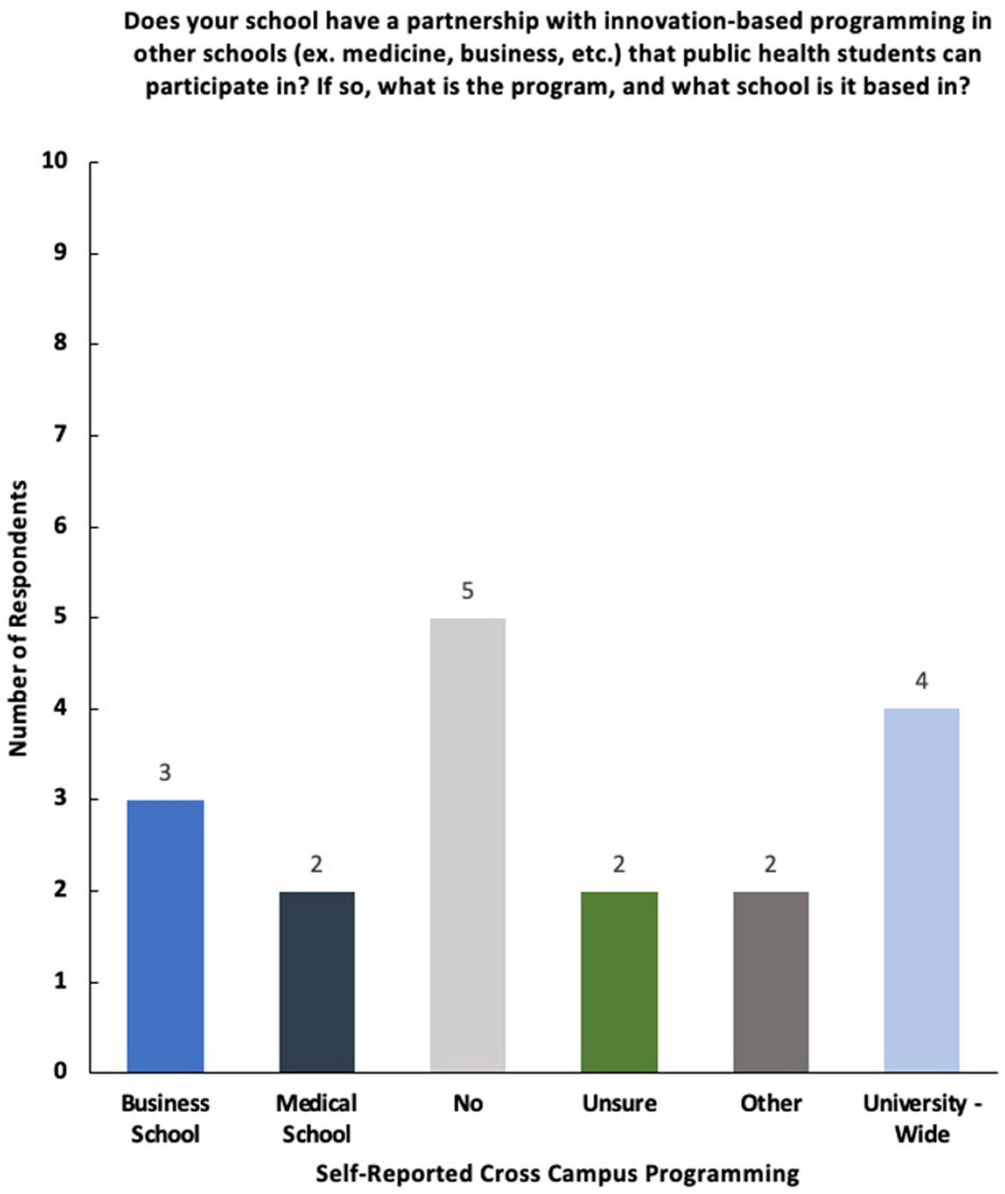
Breakdown of responses to the survey question—“Does your school have a partnership with innovation-based programming in other schools (ex. medicine, business, etc.) that public health students can participate in? If so, what is the program, and what school is it based in?”

Overall, respondents mentioned the existence of cross-campus collaborations surrounding innovation, including collaborations between their schools of public health and schools of business and medicine, as well as university-wide initiatives (Figure 8). Many noted university-wide centers, resources, and competitions as well as collaborations between schools of public health and other schools on campus, such as schools of business and nursing. One school reported having a graduate certificate in public healthinnovation.

**Figure 9:**
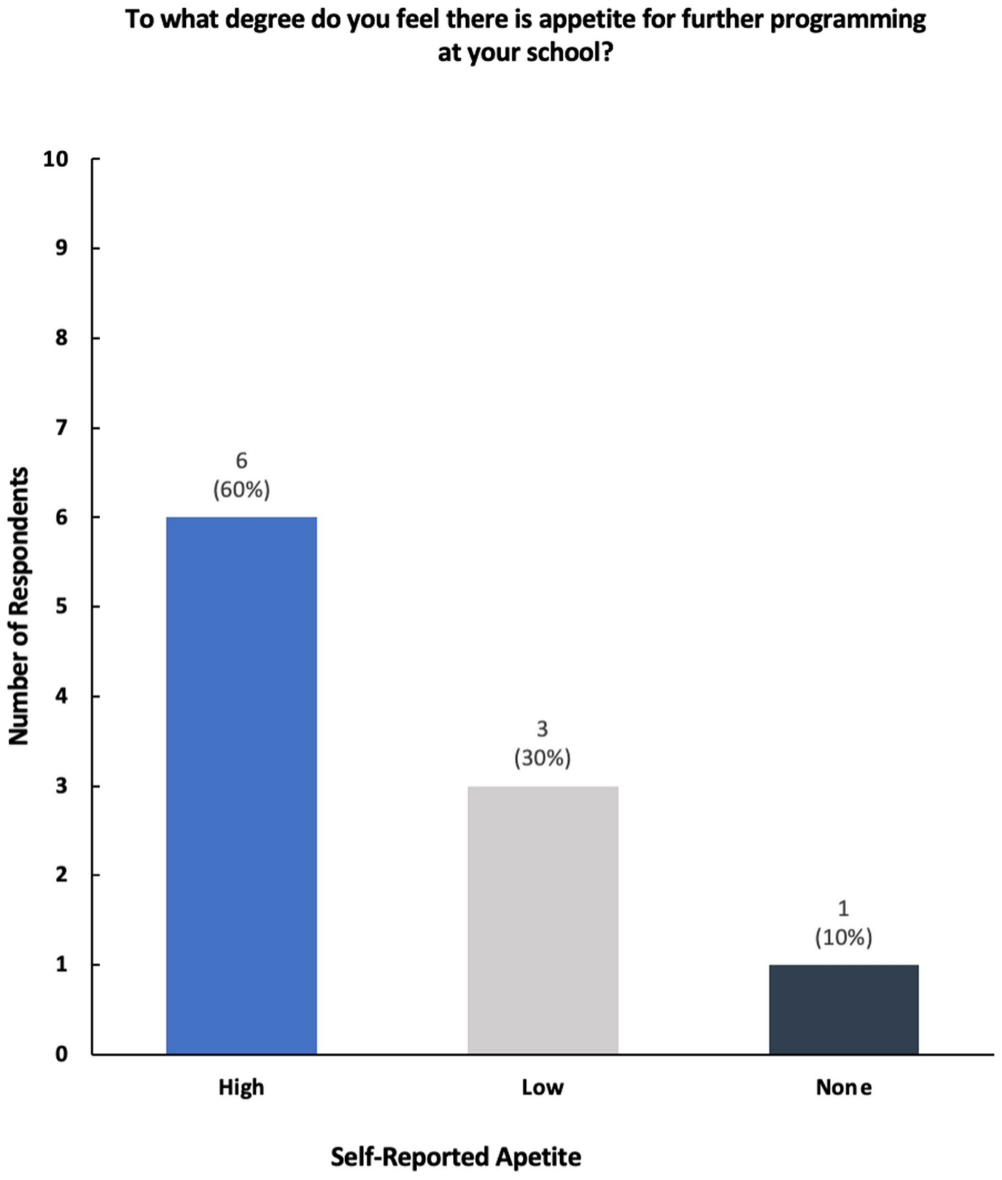
Breakdown of self-reported apetite for further innovation-based programming.

Survey respondents expanded on the future landscape of innovation and social entrepreneurial-based programming and coursework in the future. While some noted their desire to instate multidisciplinary incubators, accelerators, and innovation hubs within their schools of public health, the majority noted that they were uncertain about the creation and/or sustainability of current programming (Figure 9).

The survey also captured the insights of two public health professors outside of the United States. One professor’s experience highlights a common trend—the limited pursuit of further innovation-based programming. This too is largely attributed to shared challenges, such as insufficient funding and the perceived feeling that more immediate and pressing topics should be taught within schools of public health. These parallel observations underscore the global nature of the barriers faced in advancing health innovation and entrepreneurship education.

## Discussion

The survey results offer valuable insights into the landscape of social innovation and entrepreneurship programming within schools of public health. For instance, the survey reveals a dynamic cross-campus collaboration environment, with professors and staff members reporting a variety of innovation-based programming and collaborations between schools of public health, business, and medicine, among others; this aligns with literature emphasizing the benefits of interdisciplinary approaches in fostering innovation within public health (6).

Previous research has delved into this broader field of public health innovation and entrepreneurship, such as observing practitioners within public health ventures or departments of public health; however, the focus on specific offerings within schools of public health has received comparatively less attention (7–9). Previous studies on the field include writing commentaries, observing focus groups, and reviewing teaching frameworks, but to our knowledge, educators and staff members of public health schools have not been surveyed.

Utilizing a cross-sectional survey design allows for a snapshot analysis of the present state of social innovation and entrepreneurship initiatives, providing a timely and relevant assessment of current sentiments and efforts within schools of public health; the study collects insights from 19 professionals across 15 schools of public health, offering a nuanced understanding of the subject across various institutions.

The study identifies critical challenges faced by public health schools, such as insufficient funding, a lack of faculty engagement, and competing priorities; informing discussions on potential solutions and areas for improvement in advancing health innovation and entrepreneurship education. By exploring the availability and accessibility of student-facing programs, the study bridges the gap between academic initiatives and real-world practice, enhancing the practical relevance of the findings for public health professionals and educators. Collectively, these strengths contribute to the depth and credibility of the study, offering valuable insights for both academia and the broader public health community.

This report’s constraints involve a restricted sample size, consisting of self-selected professors from a select few universities, overlooking the comprehensive spectrum of public health entrepreneurship and innovation present in other academic settings. The limited response rate may introduce selection bias, as those who chose to participate may differ systematically from those who did not respond. We acknowledge the limitations imposed by the response rate on the generalizability of our findings; nonetheless, these findings can be insightful for other schools and programs in the field of public health seeking to integrate innovation and entrepreneurship into their curriculum.

### Recommendations

A prominent observation is the prevalence of resources available primarily to graduate students within the surveyed schools of public health. This study proposes the potential expansion of innovation-based programming for undergraduate students, including through minors and certificates. For graduate students, the desire may be for schools of public health to serve as a “hub” rather than a “spoke” of innovation on campus. Directions for future research may involved surveying public health students rather than faculty and administrators.

A noteworthy avenue for future research involves delving into the differences between curricula and programming in private versus public schools of public health. Uncovering potential distinctions in resources, course offerings, and structural support for innovation-based education can provide nuanced perspectives on the educational landscape (10). Additionally, exploring how geographic and sociodemographic factors influence the availability and effectiveness of innovation-focused programs offers a deeper understanding of regional variations and potential disparities.

Lastly, while this study focused on U.S. schools of public health, investigating the challenges and opportunities faced by international institutions can provide crucial insights into the evolution of public health curricula on a global scale. As the field of public health continues to evolve, understanding the unique constraints (or lack of) faced by diverse educational institutions trying to implement health innovation curricula is key in shaping the future of public health education. Such research can lead to more effective strategies and collaborative efforts to enhance the preparation of the next generation of public health professionals—and foster innovation and entrepreneurship within healthcare on an international scale in the process.

## Conclusion

Upon interpreting the results of this survey, it is concluded that faculty members within schools of public health have dedicated time, effort, and resources to ensuring students are at the very least familiar with social innovation and entrepreneurship and how it can be applied to improving healthcare (Figure 4). While these topics have been introduced in public health classrooms, many respondents have named faculty engagement, expertise, and funding as primary areas of focus in further building out more structured programming.

## Data availability statement

The original contributions presented in the study are included in the article/supplementary material, further inquiries can be directed to the corresponding author.

## Ethics statement

Written informed consent was obtained from the individual(s) for the publication of any potentially identifiable images or data included in this article.

## Author contributions

IH: Conceptualization, Data curation, Formal analysis, Investigation, Methodology, Resources, Visualization, Writing – original draft, Writing – review & editing. KK: Conceptualization, Data curation, Formal analysis, Funding acquisition, Investigation, Methodology, Project administration, Resources, Software, Supervision, Validation, Writing – review & editing. TC: Conceptualization, Data curation, Formal analysis, Investigation, Methodology, Writing – review & editing. FB: Conceptualization, Data curation, Funding acquisition, Investigation, Project administration, Resources, Software, Supervision, Validation, Writing – review & editing.

## References

[1] Obserg S. Martin R. (2015). *Two keys to sustainable social Enterprise.* Harvard Business Review. Available at: https://hbr.org/2015/05/two-keys-to-sustainable-social-enterprise.

[2] Organization for Economic Co-operation and Development. (2023). *Social Innovation*. Available at: https://www.oecd.org/regional/leed/social-innovation.htm.

[3] Phills J. Deiglmeier K. Miller D. (2008). *Rediscovering social innovation*. Stanford Social Innovation Review. Available at: https://ssir.org/articles/entry/rediscovering_social_innovation#

[4] Becker ERB Chahine T Shegog R. Public health entrepreneurship: a novel path for training future public health professionals. Front Public Health. (2019) 7:89. doi: 10.3389/fpubh.2019.00089, PMID: 31106187 PMC6499151

[5] Hernández D Carrión D Perotte A Fullilove R. Public health entrepreneurs: training the next generation of public health innovators. Public Health Rep. (2014) 129:477–81. doi: 10.1177/003335491412900604, PMID: 25364047 PMC4187288

[6] Chahine T. Toward an understanding of public health entrepreneurship and intrapreneurship. Front Public Health. (2021) 9:593553. doi: 10.3389/fpubh.2021.593553, PMID: 33898370 PMC8062749

[7] Jacobson PD Wasserman J Wu HW Lauer JR. Assessing entrepreneurship in governmental public health. Am J Public Health. (2015) 105:S318–22. doi: 10.2105/AJPH.2014.302388, PMID: 25689182 PMC4355718

[8] Erwin PC Brownson RC. The public health practitioner of the future. Am J Public Health. (2017) 107:1227–32. doi: 10.2105/AJPH.2017.303823, PMID: 28640683 PMC5508141

[9] DeSalvo KB O'Carroll PW Koo D Auerbach JM Monroe JA. Public health 3.0: time for an upgrade. Am J Public Health. (2016) 106:621–2. doi: 10.2105/AJPH.2016.303063, PMID: 26959263 PMC4816012

[10] Huang TTK Ciari A Costa SA Chahine T. Advancing public health entrepreneurship to Foster innovation and impact. Front Public Health. (2022) 10:923764. doi: 10.3389/fpubh.2022.923764, PMID: 35692320 PMC9184719

